# Cyclic Acetals as Novel Long-Lasting Mosquito Repellents

**DOI:** 10.1021/acs.jafc.2c05537

**Published:** 2023-01-17

**Authors:** Immacolata Iovinella, Alessandro Mandoli, Cristina Luceri, Mario D’Ambrosio, Beniamino Caputo, Pietro Cobre, Francesca Romana Dani

**Affiliations:** † Biology Department, University of Firenze, via Madonna del Piano 6, 50019Sesto Fiorentino, Italy; ‡ Department of Chemistry and Industrial Chemistry, University of Pisa, Via G. Moruzzi 13, 56124Pisa, Italy; § NEUROFARBA Department, Section of Pharmacology and Toxicology, University of Firenze, Viale Gaetano Pieraccini 6, 50100Firenze, Italy; ∥ Department of Public Health & Infectious Diseases, University “La Sapienza”, Piazzale Aldo Moro 5, 00185Roma, Italy

**Keywords:** acetal, Aedes albopictus, protection time, olfaction, toxicity, vector-borne diseases

## Abstract

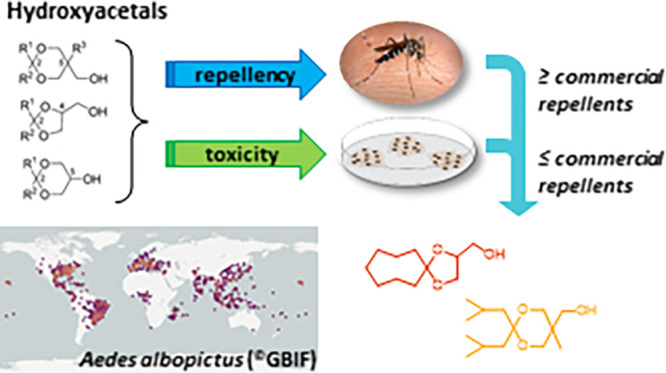

The use of skin repellents against hematophagous mosquitoes is an important personal protection practice wherever these insects are abundant and where they are vectors of diseases. DEET and Icaridin are the major synthetic insect repellents in commercial formulations and are considered the most effective. Here, we tested against the mosquito *Aedes albopictus* several cyclic hydroxyacetals synthesized by acetalization of commercially available aliphatic carbonyl compounds (ranging from C3 to C15) with either glycerol, 1,1,1-trismethyloletane, or 1,1,1-trismethylolpropane and compared their efficacy with commercial repellents. We found that several hydroxyacetals were comparable with DEET and Icaridin both in terms of the required dose and repellence duration, while a few performed better. For those most active, toxicity was investigated, finding that a few of them were less cytotoxic than DEET and less prone to permeate through cell layers. Therefore, such results indicate that novel safe mosquito repellents could be developed among cyclic hydroxyacetals.

## 1 Introduction

**Figure 1:**
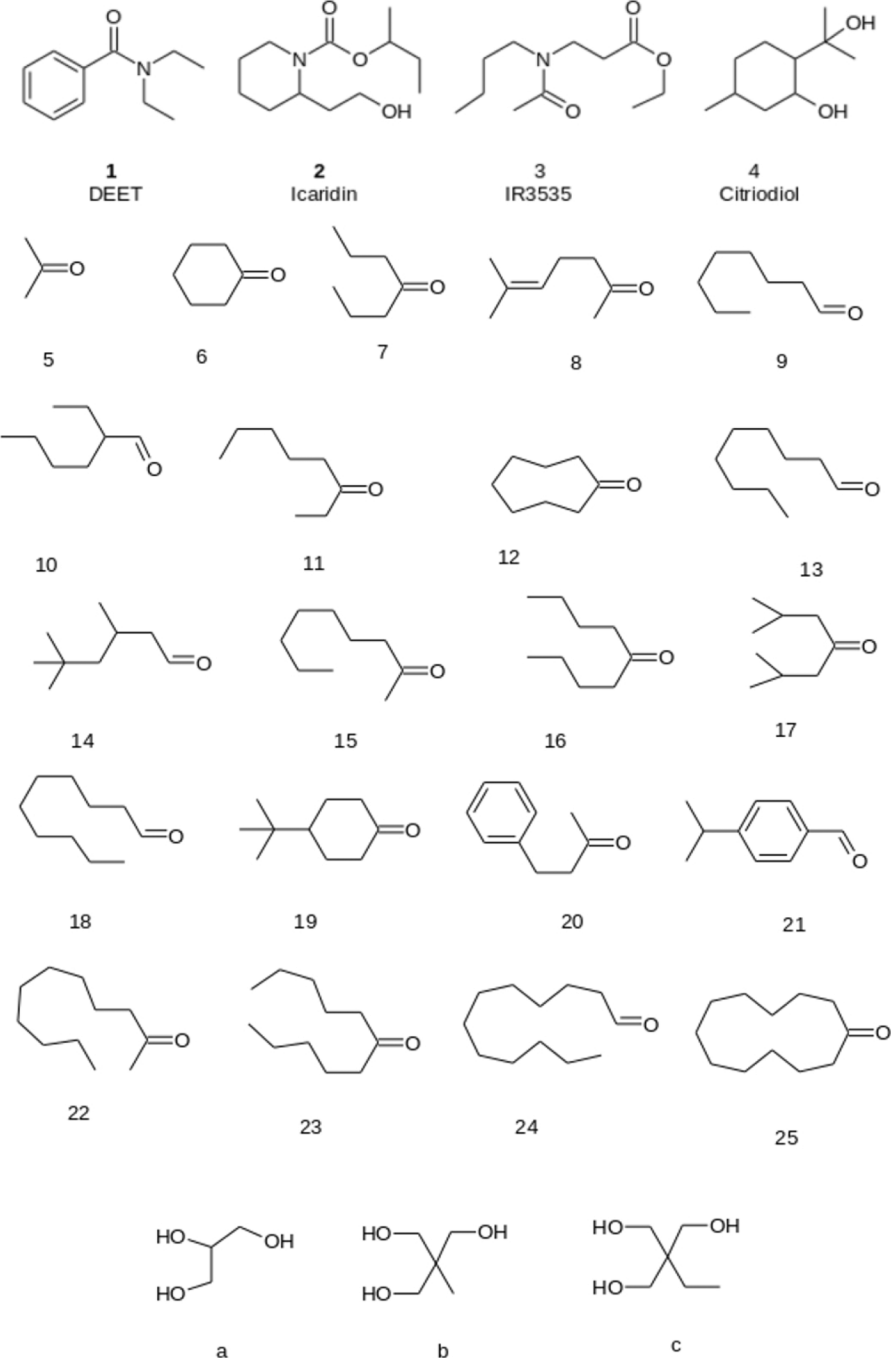
Common commercial mosquito repellents (**1–4**) and precursors to the cyclic hydroxyacetals examined in this work (**5–25**: carbonyl compounds and **a–c**: triols).

Mosquitoes are responsible for spreading several serious diseases, accounting each year for 2.7 million deaths worldwide, mainly in developing countries. Some of the most actively investigated approaches to keep mosquitoes away from humans focus on the signals they use to locate hosts and how to disrupt such information using repellents. Among the major topical repellents on the market, DEET was regarded until recently as the golden standard for this approach, despite several limitations such as its odor, the need for frequent application, and high-concentration formulations that damage synthetic fabrics and plastics.^1,2^ Icaridin is now regarded as a better alternative, having been classified as practically non-toxic, not likely to be carcinogenic by the dermal route,^3^ and more cosmetically pleasant to use.^2^ DEET and Icaridin (Figure 1) show a dose-dependent effect: the higher the concentration, the longer the protection. Accordingly, Icaridin-based formulations are available in concentrations from 5 to 20%,^4^ while in commercial formulations, the concentration of DEET may range from 4% to nearly 100%.^5^ Alternatives to these two chemicals are the natural compound *p*-menthane-3,8-diol (Citriodiol and the synthetic amide ethyl butylacetylaminopropionate (IR3535) (Figure 1), both possessing favorable cosmetic characteristics, but the former product has a shorter general persistence than DEET, while the latter is not recommended in countries where malaria is endemic.^1^

### 1.1 Synthetic Mosquito Repellents

After the commercialization of the first major synthetic insect repellent DEET (*N*,*N*-diethyl-3-methylbenzamide), for many decades, the search for efficient mosquito repellents has followed a structure–activity approach. Based on the structures of DEET and Icaridin, several studies have tried to dissect the structural elements important to elicit repellence and introduce minor modifications to obtain products with improved features.^6−9^ None of these attempts has succeeded in discovering repellents more efficient than DEET and Icaridin. Nevertheless, such studies provided valuable information for designing compounds that might offer longer protection time and reduced toxicity or could be easier and cheaper to synthesize, while being as effective as DEET and Icaridin.

Following a different approach, other studies have searched for repellents spanning a wide variety of chemical structures and functional groups.^10^ The most striking observation is that efficient repellents belong to very diverse chemical classes, from benzoates and phthalates to diols, such as 2-butyl-2-ethyl-1,3-propanediol and 1-propyl-2-propyl-1,3-pentanediol, or amides, such as ethyl butylacetylaminopropionate (IR3535).^11−18^

### 1.2 Development of New Mosquito Repellents

At present, DEET, Icaridin, IR3535, and the natural citriodiol have been registered as topical repellent ingredients.^19,20^ None of these four commercial repellents (Figure 1) embodies on their own all the ideal features of a skin repellent: highest repellent activity at low dose; negligible acute and chronic toxicity for humans, animals, and the environment; negligible or faint and pleasant odor; non-greasy feeling on the skin; resistance to abrasion from clothing, evaporation, and absorption from the skin surface; wash-off from sweat or rain; and ease of formulation in a water medium.

In two previous papers,^21,22^ we took a different approach to design mosquito repellents and modified the structure of two natural terpenoid repellents. Since most naturally occurring terpenoids endowed with an insect repellent activity show a short protection time against hematophagous insects, mainly because of their volatility, we hypothesized that derivatives of two well-known terpenoid repellents, menthone and citronellal, with lower volatility would have a longer protection time. This was confirmed by converting the starting compounds into cyclic acetals and hydroxyacetals through condensation with diols and with glycerol; in both cases, the protection time exceeded that of DEET.

In this work, we followed a similar approach using different triols to synthesize cyclic hydroxyacetals. The presence of a free hydroxyl group decreased the volatility of the final derivatives and drastically reduced their odor, while the improved hydrophilicity allowed for easier formulations in aqueous media. Moreover, we have expanded the choice of our chemicals including other carbonyl compounds which were not reported as mosquito repellents. Some other compounds are known as attractants, being constituents of the odorant profiles of mosquitoes hosts, such as octanal, nonanal, and decanal,^23^ but at certain concentrations and under some conditions have also a repellent activity.^24−27^ Notwithstanding, most of the novel cyclic hydroxyacetals were also found to be efficient repellents, thus suggesting that such activity might be linked to the cyclic acetal moiety. Besides testing repellence activity and comparing it with those of Icaridin and DEET, we deemed it crucial to perform a preliminary evaluation of the safety of this class of acetals for which no biological data are available.

## 2 Materials and Methods

### 2.1 Materials

All the starting materials (Figure 1) were purchased either from Sigma-Aldrich, Carlo Erba (4-heptanone, cyclohexanone; 6-methyl-5-hepten-2-one), or Fluka (diisobutylketone) and used as received. The technical grade of diisobutylketone (compound **17**) was found by gas chromatography–mass spectrometry (GC–MS) to consist of a mixture of **17** and 4,6-dimethyl 2-heptanone in an approximately 3:1 ratio.

### 2.2 Synthesis and Characterization of the Cyclic Hydroxyacetals

The candidate repellent compounds examined in this study were obtained by the acid-catalyzed condensation of the triols and carbonyl components, following one of the three general methods (A–C) described in the Supporting Information (File S1).

Each product was analyzed by GC–MS on a 7820 GC system coupled to a 5977B MSD (single quadrupole, Agilent Technologies). Separation was made on a 19091S-433UI column (stationary phase, 95% PDMS, 5% benzene; 30 m × 0.25 mm, Agilent Technologies), using helium as carrier gas (1 mL min^–1^) at 45 °C (2 min); 10 °C min^–1^ up to 200 °C (3 min); and 15 °C min^–1^ up to 300 °C (2 min). The injector port was set at 250 °C. Solutions (1 μL, 50–200 ng) of each product were injected.

Electronic ionization was set at 70 eV and acquired *m*/*z* ranging from 50 to 550. Data were analyzed using the software Agilent MassHunter Qualitative Analysis B.07.00, and spectra were checked for diagnostic ions expected based on the product structures. In the syntheses where both 1,3-dioxane and 1,3-dioxolane isomers were expected, the latter could be identified for the more intense ion at [M-31]^+^, due to the loss of the CH_2_OH fragment.^28^

If not noted otherwise, the NMR spectra were recorded in CDCl_3_ at room temperature with a Bruker AVANCE DRX 400 spectrometer (401.36 MHz for ^1^H and 100.93 MHz for ^13^C). For referencing the chemical shift scale (δ), the resonances of the not deuterated residual solvent (^1^H) or the deuterated solvent (^13^C) were set to the recommended values.^29^ Due to the appreciable acid sensitivity of some of the hydroxyacetals, the CDCl_3_ employed for recording the NMR spectra was stored over K_2_CO_3_ and filtered through a short pad of the same basic agent just before use. Even so, especially in the case of ketone derivatives, some degradation of the product was occasionally observed upon dissolution in CDCl_3_. To circumvent the problem, a few spectra were recorded in C_6_D_6_.

### 2.3 Prediction of Selected Physicochemical Properties

Estimates of the octanol–water partition coefficient (log *P*), polar surface area (PSA), and vapor pressure at 25 °C (log VP) of the hydroxyacetal included in this study were calculated with ChemBrain IXL (vers. 5.9), database and prediction software developed by Naef and Acree.^30^ The results of these calculations are summarized in Table B (File S1).

### 2.4 Effective Dose of Synthesized Compounds as Mosquito Repellents

The repellence of DEET, Icaridin (both supplied by Istituto Biochimico VEBI s.r.l.), acetals, and the synthesized hydroxyacetals was evaluated against*Aedes albopictus*using the human-bait technique (to simulate the condition of human skin on which repellents will be applied).^22^*A. albopictus*was reared and tested at 26 ± 2 °C, ≥60 ± 10% relative humidity (RH), and at 14:10 h light/dark photoperiod, within Plexiglas cylindrical laboratory cages (35 cm diameter, 60 cm length) with one end closed by a net. During the tests, cages contained ≈150 nulliparous, 4–7 day-old, nonblood-fed females. For each compound to be tested, up to six volunteers participated in the trial.

The study was approved by the Regional Ethics Committee for Clinical Trials of Tuscany Region with the registered number 20383. Volunteers agreed to take part in the experiments following informed consent; only volunteers non-allergic to mosquito bites participated in the trials. On the day of the bioassay, they had no contact with lotions, perfumes, oils, or perfumed soaps. They wore latex surgical gloves, in which a dorsal square area of 30 cm^2^ was cut open. Mosquito-exposed skin was treated with 50 μL of ethanol, as the negative hand control. The other hand was treated with 50 μL of the tested compounds in ethanol solution (dosages corresponding to 0.081, 0.17, 0.33, 0.5, 0.83, 1.7, 8.3, 16.7, and 83.3 μg/cm^2^). Both hands were presented in the same test cage. The number of probing mosquitoes in a 3 min exposure period was recorded. During each test, the control and the treated hand were presented interchanged to verify the mosquitoes’ readiness to bite. Trials were considered valid only if at least 30 mosquitoes performed probing behavior on the control hand before each repellent dosage was tested. The protection efficacy (PE %) obtained from all replicates was calculated, according to the WHO guidelines^31^ using this formula
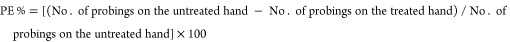


### 2.5 Protection Time of the Synthesized Compounds

To evaluate the protection time of derivatives, the PE % was measured with 100 μL of a 5% ethanol solution, corresponding to 0.17 mg/cm^2^ of exposed skin, under the same laboratory conditions. Mosquito-exposed skin was treated with 100 μL of ethanol, as the negative hand control. For each volunteer (up to 8), the test was performed every hour, up to 8 h from the application. The protection time of DEET and Icaridin was measured under the same conditions. Since these trials were run in parallel with those aimed to profile the cytotoxicity of the compounds (paragraph 2.6), we interrupted the experiments for those compounds showing to have higher cytotoxicity than Icaridin and DEET as soon as the results were available. For this reason, the number of volunteers differs among the compounds.

The average time until protection which was higher than 95% protection time was considered for each compound. Moreover, for the compounds tested on at least three volunteers, the complete (100%) protection time was also estimated (SPSS 28.01.0) using the Kaplan–Meier survivor function procedure.^22^

### 2.6 Toxicity Profile of the New Derivatives

#### 2.6.1 Cytotoxicity on Human Keratinocytes

To test the effect of the new derivatives on cell integrity, normal human keratinocytes (HaCaT cells, from ATCC, USA) were used to measure cell viability after exposure to the most promising compounds, using DEET and Icaridin as references. HaCaT cells were cultured in 5% CO_2_ at 37 °C in Dulbecco’s modified Eagle’s medium (DMEM), supplemented with 10% fetal bovine serum (FBS), 1% l-glutamine (4 mM), and 1% penicillin–streptomycin (Thermo Fisher Scientific, Rodano, Milan, Italy). HaCaT cells were then seeded (1 × 10^5^ cells/well) and exposed to 10 derivatives (**6b**, **9b**, **12a**, **13a**, **15a**, **16b**, **16c**, **17b**, **17c**, and **18a**) or to DEET and Icaridin, at a final concentration of 82 μg/mL, selected to obtain a final solvent concentration (ethanol) below 0.1%. Cytotoxicity was evaluated after 24 h using the MTS reagent (CellTiter 96 Aqueous proliferation assay; Promega Madison, WI, USA) as previously described.^32^ For compounds exhibiting cytotoxicity at 24 h but of interest for their repellence, we evaluated also the HaCaT viability after 3 and 6 h of exposure. Experiments were performed in triplicate.

#### 2.6.2 Transwell Permeation Test

To evaluate the possibility of absorption after topical application, we tested the permeation through a Caco-2 monolayer for those compounds best performing in terms of protection time and cytotoxicity. The colorectal adenocarcinoma cell line Caco-2 was purchased from ATCC and cultured in DMEM with 20% FBS, l-glutamine (2 mM), and 1% penicillin–streptomycin 100 U/mL (Thermo Fisher Scientific, Rodano, Milan, Italy) in 5% CO_2_ at 37 °C.

For the permeation studies, Caco-2 cells, a model of epithelial cells, were seeded into 12-well PET Transwell plates (1.13 cm^2^ growth surface area and pore size 0.4 μm, Greiner Bio-One, Milan, Italy) at a density of 2 × 10^5^ cells/cm^2^ and grown for 21 days to form a confluent monolayer. The integrity of the cellular barrier was then assessed using Lucifer Yellow (LY) permeability test, before the experiments.^33^

After washing, the monolayers were preincubated for 20 min at 37 °C with 0.5 and 1.5 mL of the incubation medium, HBSS/HEPES 25 mM in the apical and basolateral sides, respectively. After preincubation, the medium was removed immediately, and the incubation medium containing new derivatives or Icaridin at the same concentration tested on HaCaT cells was added to the apical side. After 3 h, 500 μL of media from the basal compartment of each Transwell plate was collected. At the end of the experiment, the layer integrity was re-evaluated. Experiments were performed in triplicate.

One hundred microliters of the collected media was extracted in 100 μL of heptane by vortexing the vial for 2 min; 70 μL of this solution was recovered; and 1 μL was injected in a 7820 GC system-5977B MSD system and analyzed under the same conditions reported in paragraph 2.2.

While peaks could be easily integrated for compounds **16b**, **17b**, and Icaridin, when spectra were acquired under full scan conditions (*m*/*z* 50–550), analyses of compounds **12a** and **17c** were acquired under selected ion monitoring (SIM) conditions by targeting the most intense ions (*m*/*z* 185, 157, 138, 129, and 116 and the molecular ion 200 for **12a**; 201, corresponding to M-57, 143, and 85 for compound **17c**). Calibration curves, to be applied to estimate the analyte concentration, were calculated by extracting and analyzing, using the same methods, 100 μL of the HBSS/Hepes solutions at the following concentrations: 1.6, 3.3, and 8.2 μg/mL.

Each analysis was performed in triplicates.

#### 2.6.3 Immunogenicity on Murine Macrophages

To explore the potential immunogenicity of the compounds best performing in terms of repellency, cytotoxicity, and low permeation, we analyzed the ability to activate RAW 264.7, a murine macrophage cell line obtained from ATCC. Cells were cultured in DMEM, with 10% FBS, 1% l-glutamine (2 mM), and 1% penicillin–streptomycin 100 U/mL at 37 °C in an atmosphere containing 5% CO_2_. RAW264.7 were seeded (1 × 10^5^ cells/well) and exposed to five derivatives or Icaridin, at 82 μg/mL or to LPS (1 μg/mL) as a positive control, for 3 and 6 h. After that, nitric oxide release was determined in the culture media as previously described.^32^ Experiments were performed in triplicate.

For all the experiments, a Kruskal–Wallis test and Dunnett’s multiple comparisons test were performed through the software GraphPad Prism 7.

## 3 Results and Discussion

### 3.1 Synthesis and Chemical Characterization of the Hydroxyacetals

Starting from the observation that the acetalization of citronellal and menthone with glycerol still preserves the repellent activity of parent monoterpene carbonyl compounds while increasing their protection time,^21,22,34^ we extended the investigation to several cyclic hydroxyacetals resulting from the condensation of cheap and commercially available carbonyl compounds (Figure 1, **5**–**25**), with glycerol (**a**), 1,1,1-trimethylolethane (**b**), or 1,1,1-trimethylolpropane (**c**). Irrespective of its actual composition (vide infra), each synthesized product is named in the following as **nx**, where **n** and **x** are the number and the letter of the starting carbonyl and triol precursor, respectively.

One reason behind the selection of the polyhydroxylated alcohols **a**–**c** was to investigate only cyclic acetals (1,3-dioxolanes and 1,3-dioxanes) because of their expected higher stability as compared to open-chain acetals. At the same time, with this choice, the resulting products could be endowed with a free hydroxy group that, besides mimicking the polar side chain of Icaridin (**2**), was expected to reduce the volatility of the acetals and ease their formulation in water. Also, the two donor oxygen atoms and the hydroxy group featured in the designed compounds were expected to be favorable structural features, since Icaridin is also a bifunctional compound featuring a donor atom and a hydroxy group. Triols **a**–**c** were adopted for their different contributions to the lipophilicity of the respective acetals.

**Figure 2:**
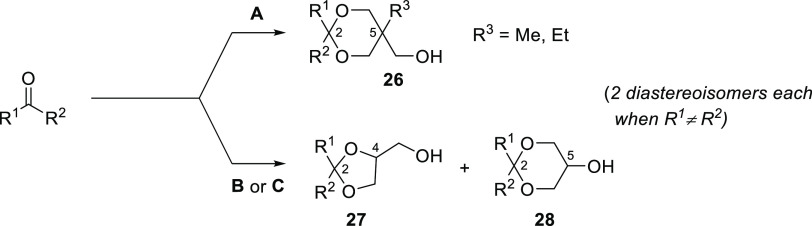
Preparation of the hydroxyacetals **26**, **27**, and **28**. Reagents and conditions. (A) (i) MeOH, HC(OMe)_3_, cat. TsOH; (ii) R_3_C(CH_2_OH)_3_, 60 °C. (B) Glycerol, aldehyde, cat. Amberlyst 15, EtOH, 60 °C. (C) Glycerol, *n*-hexane, cat. TsOH, reflux with azeotropic removal of water (Dean–Stark).

Among the many procedures reported to date for the acetalization of carbonyl substrates with polyhydric alcohols (for a review of earlier achievements, see ref^35^ and references therein), three general synthetic methods were exploited in this study for the preparation of the candidate repellents (scheme in Figure 2): the trans-acetalization reaction between trimethylolethane or trimethylolpropane and the methyl acetal of the carbonyl precursor (method A), the direct reaction between an aldehyde and glycerol, in the presence of the sulfonated polystyrene resin Amberlyst 15 (method B), or the reaction of a ketone with glycerol, in the presence of *p*-toluensulfonic acid (TsOH) and with azeotropic removal of water (method C).

All the procedures could be easily scaled-up to the >100 g size and afforded the hydroxyacetals in >90–95% yields and satisfactory purity (90 to > 99%, see Table A in File S1; for example, respectively, 90 and 95% for **12a** and **17b**), after simple work-up. Altogether, 27 derivatives were synthesized, whose structures, selected NMR constants, GC retention indexes, and purity are listed in Table A of File S1. All the products were obtained as clear, colorless to light-yellow oils, with faint, pleasant odors, much weaker than their carbonyl precursors.

As expected from previous investigations,^35−37^ the GC–MS and NMR analyses of the products obtained from glycerol revealed the formation of several cyclic products, whose actual distribution was found to depend on the nature of the starting carbonyl material. In detail, although ketones gave the five-membered cyclic derivative (1,3-dioxolane, Figure 2, general structure **27**) in a nearly exclusive manner, the aldehydes provided a mixture of the former and its six-membered isomer (1,3-dioxane, general structure **28**) in comparable amounts. Thanks to the equivalence of the three hydroxylated arms, this problem did not arise using trimethylolalkanes as the polyhydric components. Nevertheless, whenever R1 ≠ R2 (aldehydes and non-symmetrically substituted ketones), the presence of two stereogenic centers within the saturated heterocyclic cores (C2 and either C4 or C5) led to the obtainment of the acetal products (**26** or the mixture **27** + **28**) as cis/trans diastereomeric pairs.

Further molecular variability in the preparations arose from the use of the chiral aldehydes **10** and **14** in their racemic forms and because of the presence of approx. 25% 4,6-dimethylheptan-2-one (GC–MS) in the technical grade diisobutylketone (compound **17**) employed in this study.

No attempts were made herein to separate the single components in any of the mixtures mentioned above, the isomeric blends being used for the analytical measurements (File S1) and the repellency tests. The only exception in this respect was **18a**, which separates the crystalline *trans*-1,3-dioxane isomer (*trans*-**18a-6** in File S1), on standing at room temperature. In this case, the prompt availability of the nearly pure substance permitted to compare the bioactivity of the stereochemically defined single component with that of the whole isomeric mixture (vide infra).

### 3.2 Repellent Activity of the Synthesized Compounds

Table 1 reports a selection of hydroxyacetals with the strongest repellency activity, whereas Table S1 summarizes the full data set; the original data are reported in Table S2 (effective dose) and Table S3 (protection time). We evaluated the repellent properties of all the synthesized chemicals against adult females of *A. albopictus* and compared their activity with those of the reference commercial products DEET and Icaridin.

**Table 1:**
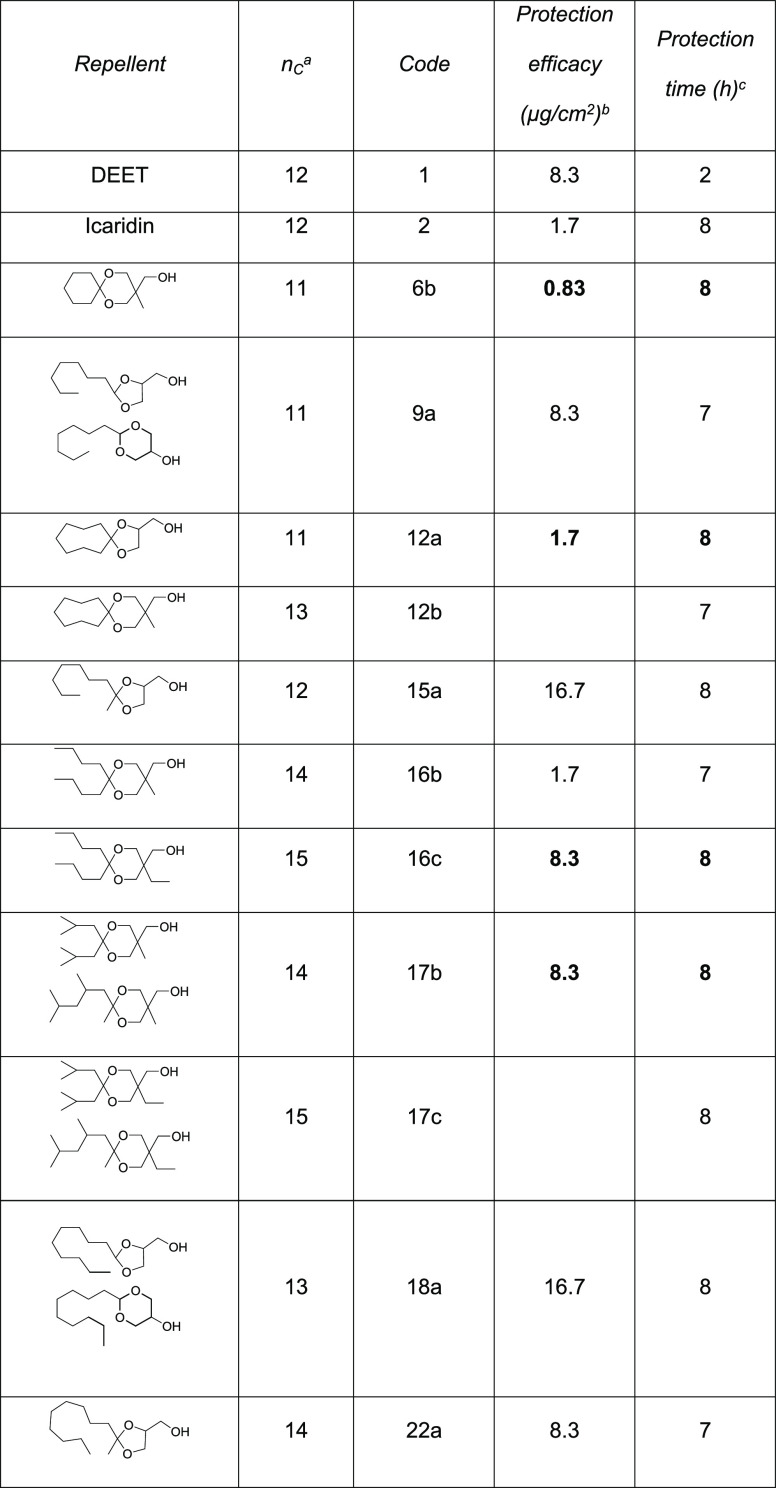
Repellency Properties of Selected Hydroxyacetals^d^

a Total number of carbon atoms in the substance.

b To obtain complete protection (100%) from bite attempts.

c 95% protection at a dose of 0.17 mg/cm^2^.

d Numbers in bold indicate a better or comparable repellence with respect to Icaridin.

To evaluate the protection efficacy, volunteers applied increasing doses of the compounds to the skin on the back of their hands (30 cm^2^, while the rest was covered with a rubber glove) and counted the number of mosquitoes attempting to bite; the solvent only was used on the control hand. The protection (percentage of repelled mosquitoes) was calculated as reported in the Material and Methods section.

Seven of our cyclic acetals can repel mosquitoes when applied on the skin in doses of 1 to 8.3 μg/cm^2^ (Table 1), which are the same measured for DEET and Icaridin under the same conditions.

**Figure 3:**
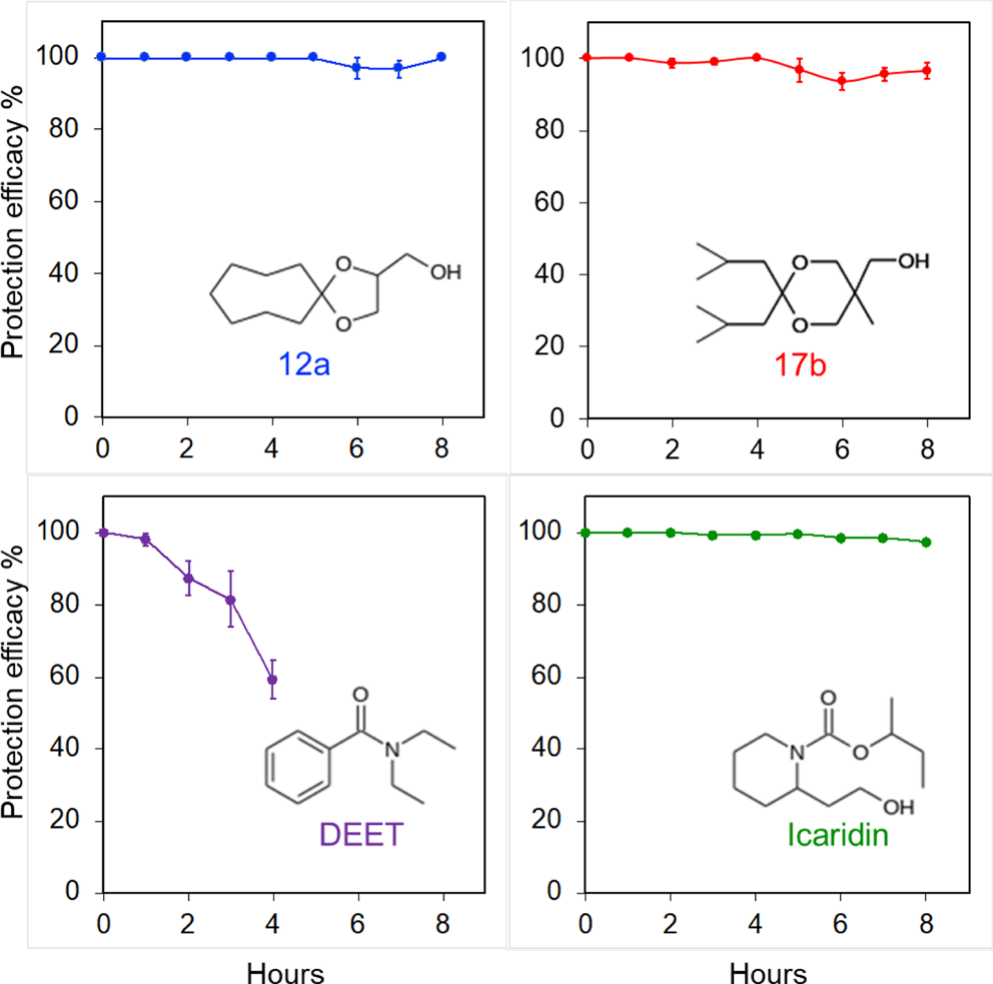
Protection efficacy (mean, SE) over time of compounds **12a**, **17b**, DEET, and Icaridin against bite attempts by*Aedes albopictus*when they were applied on the skin at a dose of 0.17 mg/cm^2^ and tested at 1 h intervals until 8 h after the application.

The protection time was measured by applying all our cyclic acetals, DEET, and Icaridin at the same dose of 0.17 mg/cm^2^ and testing them against *A. albopictus* for 8 h. We found that Icaridin and seven of our compounds kept a repellence above 95% for at least 8 h, while at the same dose, DEET was active for only 2 h (Table 1). The graphs of Figure 3 illustrate such experiments taking as examples derivatives **12a** and **17b**, as well as DEET and Icaridin. The estimated complete (100%) protection time was longer than that of DEET (average ± SE; 120 ± 26 min) for most of the compounds and comparable with Icaridin (370 ± 52 min) for a few of them (Table S1), including compound **12a** (380 ± 20 min) which was also among the best performing compounds for protection efficacy and toxicity (see below).

It is also worth noticing that for practical reasons, we ended all experiments after 8 h; therefore, we cannot exclude that some of the repellents might be active for longer times.

The results (all reported in Table S1) look very promising, although more detailed and extensive experimentation is needed.

### 3.3 Cell Toxicity

A preliminary evaluation of the safety of this class of acetals for which no biological data are available has been performed. Thus, the toxicity of the most promising repellents was evaluated in terms of cell toxicity, immunogenicity, and epithelial permeability using standard tests. DEET and Icaridin were also included in the study as reference standards.

Cell toxicity was evaluated in terms of the percentage of keratocyte survival after 24 h exposure to compound solutions.^32^ For a selection of these, immunogenicity (activation of RAW264.7 with LPS as the positive control)^32^ and epithelial permeability (transwell permeation), as described in the Material and Methods section, were finally evaluated.

**Figure 4:**
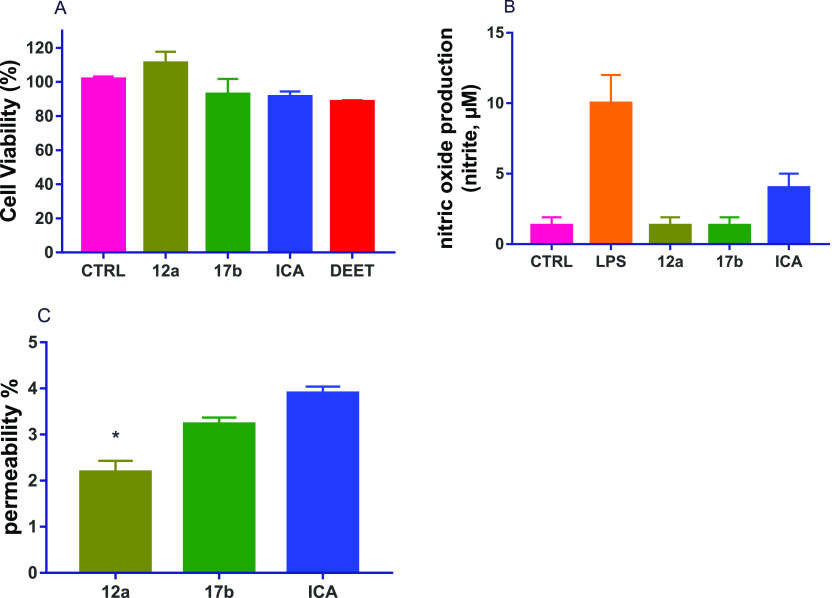
(A) Cytotoxicity on normal human keratinocytes (HaCaT) exposed to compounds **12a**, **17b**, Icaridin (ICA), and DEET tested at 82 μg/mL for 24 h; (B) immunogenicity (measured as nitric oxide release) on murine macrophages (RAW264.7) stimulated with LPS (1 μg/mL) or exposed to compounds **12a**, **17b**, and Icaridin, 82 μg/mL, for 6 h; (C) transwell permeation test, percentage of the compounds passed through a Caco2 cell monolayer. *p < 0.05 vs Icaridin (ICA) by Kruskal–Wallis test and Dunnett’s multiple comparisons test; data are expressed as average ± SE of three independent experiments.

Some of the best repellents proved also to be endowed with low cell toxicity. Values of cell survival are comparable to those of DEET or Icaridin or even better (Figure S1). In particular, four compounds (**6b**, **12a**, **16c**, and **17b**) outperformed DEET (>88% survival of keratocytes), while the cyclic ketone derivatives **12a** (100% survival) and the open-chain ketone derivative **17b** (92% survival) turned out to be even less toxic than Icaridin (91% survival) (Figure 4A). Acetals **12a** and **17b**, which emerged as the most promising from the abovementioned tests, were also evaluated in terms of immunogenicity and epithelial permeability (transwell permeation). In the former test, both compounds did not exhibit immunogenicity, while Icaridin induced a three-time increase in the nitrite production compared to the control treatment (Figure 4B). In the transwell permeation test, **12a** and **17b** exhibited permeability values of 44 and 17% lower compared to Icaridin, respectively (Figure 4C), and **12a** resulted to be significantly different from Icaridin (*p* = 0.007).

### 3.4 Structure–Activity Relationships

The repellent activity of novel compounds spans the full range from inactive to extremely active (95% protection for >8 h), thus meeting our expectations. It should be mentioned here that reference compounds DEET and Icaridin turned out to have quite different protection times (2 and 8 h, respectively), demonstrating the sharp superiority of Icaridin over DEET. Top performing acetals are evenly distributed among C11–C15 compounds, and there is no particular prevalence of any of the acetal types **a**, **b**, or **c**; however, although these incorporating glycerols (**12a**, **15a**, and **18a**) were obtained from quite structurally different carbonyl compounds (i.e., the cyclic C8 and open-chain C9 ketones and the linear C10 aldehyde), the acetals from the trimethylolalkanes gave the best repellents (**6b**, **16c**, **17b**, and **17c**) when combined with the lowest C6 cyclic or the C9 open-chain ketones. With the notable exception of the lightest hydroxyacetal (**5b**) and those featuring rigid or hindered substituents (**19a**–**21a**), most of the other derivatives with a total number of carbon atoms in the range of C11–C15 (**7a** to **17a**) are still endowed with good repellency (distinctly superior to DEET). Apart from these trends, it is not easy to recognize factors modulating the activity nor any relationship between repellence and the single-value log *P* and PSA physicochemical descriptors of the molecular properties (Table B in File S1).

For what concerns the influence of volatility, a seemingly important physical property for the efficacy of a repellent,^38^ we speculate that the lack of activity of **5b** (a C8 derivative) may be largely due to its rapid loss by evaporation. Nonetheless, when the compounds for which an estimate of saturated vapor pressure could be obtained from *ChemBrain IXL* are examined (the aldehyde-based hydroxyacetals, see Table B in File S1), again no obvious correlation emerges between the experimentally determined repellence and the predicted log VP values.

The analysis of factors affecting the bioactivity of the hydroxyacetals included in this study is further complicated by the fact that most of the tested compounds were mixtures of regio- and stereoisomers. The systematic separation of these mixtures and the investigation of their components as single substances were beyond the aims of this work. However, in the case of the decanal/glycerol acetal **18a**, the trans 1,3-dioxane component (*trans*-**18a.6**) crystallized out of the mixture and could be evaluated separately (Figure C in File S1). By these means, it was possible to conclude that *trans*-**18a.6** is inactive as a repellent and thus unable to contribute to the high repellence of the whole isomeric blend **18a**. As suggested by its longer retention time in GC, in part this lack of activity might be attributed to the lower volatility of *trans*-**18a.6** in comparison to its isomers *cis*-**18a.6** and *cis*- or *trans*-**18a.5**. Nonetheless, it should be noticed that the single component acetals **16c** and **12b** are comparable with *trans*-**18a.6** in terms of GC retention time but rank among the top performing repellents. Together with the lack of activity of the compounds provided with rigid or hindered substituents (vide supra), this comparison might suggest that the repellent effect may depend on specific interactions with the peripheral olfactory system of mosquitoes.

As to the toxicity, some of our compounds were found to be comparable with DEET and Icaridin. Compounds outperforming DEET (>88% survival) are found among those derived from symmetrical ketones (**6b**, **12a**, **16c**, and **17b**). The nature of the triol appears to have some importance on toxicity. Most noteworthily, the cyclic ketone derivative **12a** (100% survival) and the open-chain ketone derivative **17b** (92% survival) turned out to be even less toxic than Icaridin (91% survival). Acetals **12a** and **17b**, which emerged as the most promising from the tests mentioned above, were also evaluated in terms of mutagenicity (activation of RAW with LPS as the positive control) and skin permeability (transwell permeability). In the former test, both compounds turned out to be indistinguishable from the negative control, while Icaridin induced significant production of nitrite (40% as compared to the positive control). In the latter test, **12a** and **17b** exhibited 60 and 80% of permeability of Icaridin, respectively, thus proving to be both less mutagenic and less skin permeable than Icaridin.

In summary, hydroxylated cyclic acetals resulting from the condensation of readily available C6–C11 carbonyl compounds and C3–C6 triols emerge as a new class of promising mosquito skin repellents, encompassing compounds which can compete favorably with Icaridin in terms of efficacy and toxicity. Owing to the ease of preparation and possibility of formulation in water-containing media, these compounds could provide effective nitrogen-free alternatives to the most powerful active repellents present on the market. These data might be useful in providing a wider base for a better understanding of the relationship between the structure and repellent activity.

## Supporting Information Available

(File S1) Selected NMR and GC–MS characterization data (Table A and Figures A–G); predicted physicochemical properties of the hydroxyacetals (Table B); structure and repellent properties of all the synthesized hydroxyacetals (Table S1); and cytotoxicity and cell permeability of selected hydroxyacetals showing a long protection time against *A. albopictus* (Figure S1) (PDF)
(Table S2) Dose vs protection efficacy of some synthesized compounds, DEET, and Icaridin (XLSX)
(Table S3) Protection time of the synthesized compounds, DEET, and Icaridin (XLSX)
