# Correction to “Cyclic Acetals as Novel Long-Lasting Mosquito Repellents”

**DOI:** 10.1021/acs.jafc.3c05658

**Published:** 2023-08-21

**Authors:** Immacolata Iovinella, Alessandro Mandoli, Cristina Luceri, Mario D’Ambrosio, Beniamino Caputo, Pietro Cobre, Francesca Romana Dani

The word Citridiol (in line 20 of the Introduction and line 1 of section 1.2) should be replaced with eucalyptus citriodora oil, hydrated, cyclized.

