# Supplementary material for: Cyclic Acetals as Novel Long-Lasting Mosquito Repellents
Source: J Agric Food Chem. 2023 Jan 17;71(4):2152–9. doi: 10.1021/acs.jafc.2c05537 (PMC9896555; doi:10.1021/acs.jafc.2c05537)
Supplement: Supplementary file 1 — jf2c05537_si_001.pdf [file jf2c05537_si_001.pdf]

# Synthesis and characterization of the cyclic hydroxyacetals.

## Synthetic procedures

### *Method A. General procedure for acetals of trimethylolalkanes.*

A 1 L, round bottom flask was charged with *p*-toluenesulfonic acid monohydrate (1.0 g) and methanol (200 mL). While cooling in an ice bath, the carbonyl starting material (1.0 mol) was added portion-wise to the magnetically stirred solution of the acid, so as to maintain the internal temperature in the flask below 10 °C. Trimethyl orthoformate (106 g, 109 mL, 1.05 mol) was added to the resulting clear solution and the cooling bath was removed to allow warming of the mixture to r.t. Trimethylolethane or trimethylolpropane (1.05 mol) was added in one portion and the mixture was concentrated under reduced pressure (approx. 20 mmHg), while heating at 60 °C with a water bath. Sodium methoxide (5 g) was added to the residue in the flask and heating was prolonged for 15 min. After cooling to r.t., the mixture was partitioned between water (50 mL) and *n*-hexane (300 mL), and the organic layer was separated and dried over anhydrous $Na_2CO_3$. The evaporation of the solvent and other volatile components under reduced pressure (6-10 mbar) gave the product as an oil, in typically >95% yield and good GC-MS purity (Table A, compounds **05b**, **06b**, **07b**, **07c**, **09b**, **12b**, **16b**, **16c**, **17b**, and **17c**).

### *Method B. General procedure for glyceryl acetals of aldehydes.*

Dry Amberlyst® 15 (300 mm spheres, 15 mL) was washed with EtOH (50 mL), whereupon the volume of the solid increased to approx. 25 mL. The swollen resin was transferred into a solution of glycerol (94 g, 1.0 mol) in EtOH (100 g), kept in a bath maintained at 60 °C. To the manually stirred suspension, the aldehyde (0.3 mol) was added in 5 mL portions, with heating and stirring continued for one additional hour. The mixture was allowed to cool to r.t. and the catalyst beads were removed by filtration and washed with small portions of EtOH. The combined filtrates were partitioned between *n*-hexane (100 mL) and water (100 mL), and the organic layer was washed with water and brine, before drying over $Na_2CO_3$. The evaporation of the volatile components in vacuo (down to 6 mbar) gave the product as an oil (mixture of 1,3-dioxane and 1,3-dioxolane

acetals), in typically >95% yield and good GC-MS purity (Table A, compounds **09a**, **10a**, **13a**, **14a**, **18a**, **21a**, and **24a**).

## *Method C. General procedure for glyceryl acetals of open-chain or cyclic ketones.*

In a flask provided with a reflux condenser and a Dean-Stark head, a mixture of the ketone (0.2 mol), glycerol (55 g, 0.6 mol), potassium hydrogen sulfate (1.34 g, 0.01 mol) and *n*-hexane (10 mL) was heated to reflux under vigorous mechanical stirring until no more water was collected in the side-arm (approx. 8 h). The mixture was allowed to cool to r.t. and the upper hydrocarbon layer was separated from the excess of glycerol. The removal in vacuo (6-10 mbar) of the volatile components from the hexane extracts gave the product as an oil (mixture of diastereomers in the case of not-symmetrically substituted ketones), in typically >98% yield and good GC-MS purity (Table A, compounds **11a**, **12a**, **15a**, **17a**, **19a**, **20a**, **22a**, **23a**, and **25a**).

## NMR and GC-MS data

Most of the acetals examined in this work were known substances (for a list of selected references, see Tab. A). Nonetheless, for the purpose of future reference the diagnostic $^{13}C$ NMR and GC-MS features of all the single-component and mixtures of acetals compounds involved in the present study are summarized in Tab. A.

*Table A. Selected NMR and GC-MS characterization data and literature references for the acetals products.*

| Code | Structure(s) | $\delta_C$ (ppm)[a] | | GC-MS | Purity | Refs.[d] |
|---|---|---|---|---|---|---|
| | | C2 | CH-O/CH$_2$O | RI (*A*%)[b] | (%)[c] | |
| **05b** | 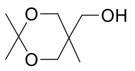 | 98.05 | 66.38, 65.79 | 11.57 | 98 | 1-2 |
| **06b** | 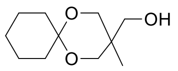 | 98.13 | 66.11, 65.58 | 15.24 | 90 | 3-5 |
| **07b** | 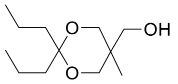 | 100.71 | 65.75, 65.60 | 15.03 | >99 | 6 |
| **07c** | 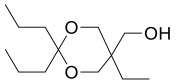 | 100.88 | 65.97, 64.61 | 16.07 | 99 | 6 |
| **09a** | 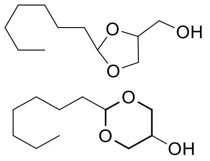 | 105.23, 104.88, 102.89, 102.10 | 76.36, 76.22, 71.78, 71.64, 66.64, 66.46, 64.14, 63.45, 62.73, 61.30 | 14.94 (19.7); 15.15 (42.4); 15.38 (20.4); 15.73 (17.5) | 92 | 7 |

| | | | | | | |
|---|---|---|---|---|---|---|
| **09b** | 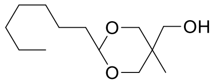 | 102.55 | 73.61, 73.01, 67.01, 65.68 | 16.88 (68.3); 17.25 (31.7) | 94 | 8-9 |
| **10a** | 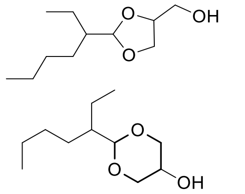 | 107.07, 107.03, 106.82, 106.80, 104.46, 103.75 | 76.24, 76.22, 76.11, 76.10, 71.83, 71.74, 66.68, 66.39, 64.21, 63.40, 62.66, 61.32 | 14.08 (30.63); 14.41 (23.21); 14.60 (21.30); 15.03 (24.86) | 97 | - |
| **11a** | 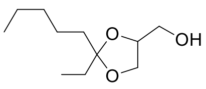 | 113.19, 113.12 | 76.45, 76.43, 66.21, 66.16, 63.12, 63.10 | 14.16 (34.29); 14.23 (65.7) | 96 | 10-11 |
| **12a** | 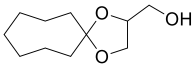 | 113.52 | 75.92, 65.53, 63.05 | 15.89 | 90 | 12-13 |
| **12b** | 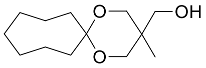 | 101.58 | 66.23, 65.76 | 17.99 | 97 | - |
| **13a** | 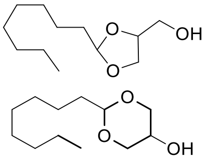 | 105.24, 104.90, 102.91, 102.11 | 76.35, 76.22, 71.79, 71.64 | 15.94 (23.8); 16.11 (29.2); 16.34 (23.5); 16.65 (23.5) | 96 | 14-15 |
| **14a** | 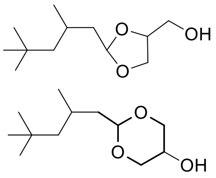 | 104.46, 104.42, 104.07, 103.93, 102.07, 101.24 | 76.28, 76.19, 76.01, 75.94, 71.86, 71.83, 71.70, 71.64, 66.57, 66.54, 66.45, 66.36, 64.21, 63.57, 63.53, 62.87, 61.42 | 14.41 (38,2); 14.67 (26,6); 14.87 (2,2); 15.16 (33,0) | 94 | - |
| **15a** | 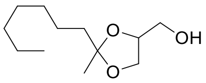 | 111.31, 111.09 | 76.62, 75.95, 65.92, 65.88, 63.14, 62.96 | 15.31 (24.25); 15.37 (75.74) | >99 | 10-11 |
| **16b** | 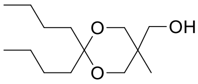 | 100.85[e] | 65.88, 65.78[d] | 16.85 | 94 | 16-17 |
| **16c** | 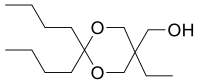 | 101.03 | 64.62, 62.94 | 17.82 | >99 | - |
| **17a** | 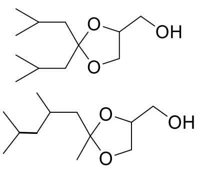 | 113.32 (111.64, 111.51)[f] | 75.97, 65.58, 63.30 (76.43, 76.26, 75.88, 75.62, 65.85, 65.78, 65.62, , 63.23, 63.14, 63.04)[e] | 13.89 (75.55); 14.22 (24.45) | 95 | 10-11, 18(-)[e] |
| **17b** | 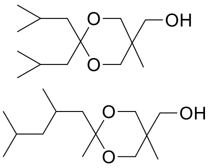 | 101.68 (100.15, 100.04)[f] | 71.97, 65.69 (66.44, 66.27, 66.08, 65.52)[f] | 15.79 (22.04); 16.05 (54.17); 16.32 (23.79) | 95 | -(-)[e] |

| | | | | | | |
|---|---|---|---|---|---|---|
| **17c** | 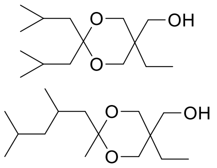 | 101.91 (100.34, 100.25)[f] | 66.46, 64.46 (64.92, 64.80, 63.32, 63.13)[f] | 16.80 (72.87); 17.16 (11.66); 17.29 (15.47) | 97 | -(-)[e] |
| **18a** | 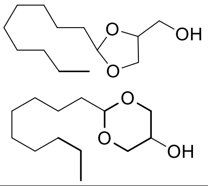 | 105.27, 104.94, 102.94, 102.14 | 76.35, 76.22, 71.82, 71.65, 66.64, 66.46, 64.19, 63.50, 62.79, 61.42 | 17.05 (20.68); 17.26 (37.16); 17.46 (23.56); 17.77 (18.59) | 99 | 19-21 |
| **19a** | 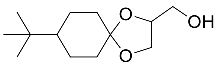 | 110.13, 110.02 | 75.90, 75.83, 65.54, 65.43, 63.30, 63.06 | 16.40 (42.25); 16.61 (55.30); 16.86 (2.45) | 99 | 22-23 |
| **20a** | 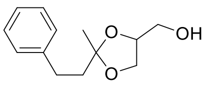 | 110.78, 110.57 | 76.82, 76.11, 66.03, 65.97, 63.13, 62.95 | 17.24 (52.98); 17.27 (47.10) | 98 | 24-25 |
| **21a** | 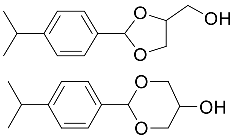 | 104.46, 103.95, 101.81, 101.08 | 76.96, 76.63, 72.34, 71.73, 66.98, 66.86, 64.11, 63.42, 62.83, 61.44 | 17.83 (22.47); 18.03 (22.88); 18.10 (20.82); 18.24 (13.81) | 99 | - |
| **22a** | 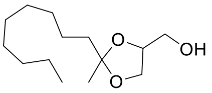 | 111.39, 111.15 | 76.60, 75.94, 65.87, 65.86, 63.18, 62.98 | 17.35 (18.53); 17.42 (81.47) | >99 | 26-27 |
| **23a** | 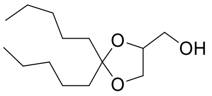 | 112.92 | 76.35, 66.09, 63.11 | 16.88 | >99 | 28-29 |
| **24a** | 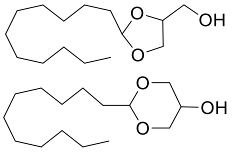 | 105.23, 104.88, 102.90, 102.10 | 76.37, 76.22, 71.76, 71.63, 66.64, 66.46, 64.12, 63.42, 62.70, 61.26 | 19.10 (25.8); 19.24 (25.4); 19.45 (19.9); 19.77 (28.9) | 94 | 21, 29-31 |
| **25a** | 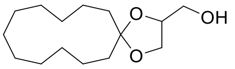 | 113.82 | 75.99, 65.58, 63.20 | 19.06 (98.89); 21.00 (1.11) | 99 | 12 |

[a] Chemical shift $\delta_C$ values of the acetal (C2) position(s) and of the other oxygenated carbon atoms in the ring or side arm of the compound(s).

[b] RI, n-Alkanes Retention Index of the acetal product(s) in the GC-MS chromatogram ([n+( Tu-Tn)/(TN-Tn)]; n = the number of carbons in the alkane preceding the compound; N= the number of carbons in the alkane following the compound; Tu = the retention time of the compound; Tn = the retention time of the preceding alkane; TN = the retention time of the following alkane); when more than one acetal compound is present, the relative peak area of each component is reported in parentheses

[c] Purity of the sample, evaluated as the (cumulative) percent area of the peak(s) assigned to the acetal(s) products with respect to all peaks present in the Total Ion chromatogram.

[d] Selected literature references reporting the preparation of cyclic acetal derivative(s) from the same carbonyl and polyol precursors.

[e] Spectrum recorded in $C_6D_6$.

[f] In parentheses, data for the minor acetal products (two or three diastereoisomers) from 4,6-dimethylheptan-2-one in technical grade diisobutylketone.

## NMR characterization of the solid hydroxyacetal isolated from 18a

NMR data for hydroxyacetals have been reported several times in the literature.14, 31-35 However, the chain of evidence that led to the reported structural assignments is not always easy to follow or rests on some early inferences about the conformational behaviour of the five- and six-membered acetal rings. To shed some light on this aspect we took advantage of the availability of the nearly pure single component that was found to crystallize from 18a upon standing. The $^{13}C$ NMR spectrum of the solid dissolved in $CDCl_3$ shows three distinct lines in the C-O region ($\delta_C$ = 102.2, 71.6, and 61.5 ppm) in approx. 1:2:1 intensity ratio. Together with the splitting patterns and 1:2:1:2 integral ratios of the resonances due to CH-O fragments in the $^1H$ NMR spectrum ($\delta_H$ = 4.39, 4.15, 3.86, and 3.35 ppm, respectively), these findings were suggestive of the 1,3-dioxane structure (**18a.6**).

Such hypothesis was substantiated by the examination of $^1H$-$^1H$ and $^1H$-$^{13}C$ scalar coupling schemes in COSY (Figure A) and HSQC experiments (Figure B), respectively.

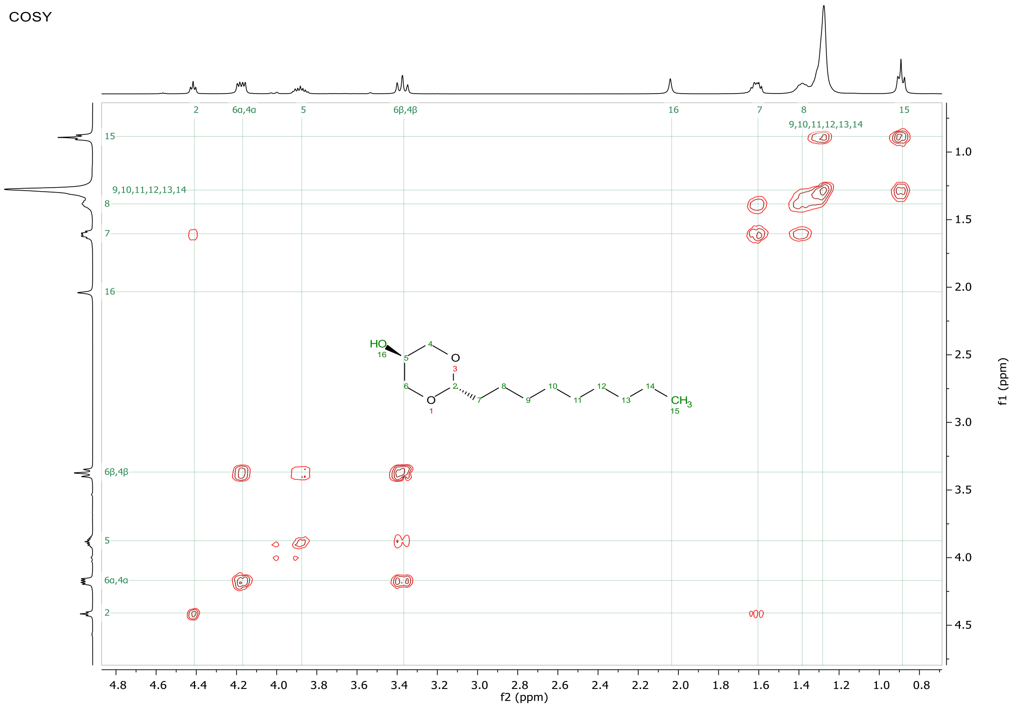


*Figure A. Gradient COSY experiment on the solid separated from **18a** ($CDCl_3$, 25°C).*

Especially diagnostic in this respect was the observation that the proton nuclei that cause the signals at $\delta_H$ = 4.15 and 3.35 ppm (2H each) provide strong cross-peaks in the COSY map and are both $^1J$-coupled (HSQC) with the carbon nuclei allied to the most intense C-O resonance ($\delta_C$ = 71.6 ppm); the latter were proved by DEPT-135 to belong to methylene fragments. Additional evidence came from a HMBC experiment (Figure C), where long-range scalar correlations between the protons resonating at either $\delta_H$ = 4.15 or 3.35 ppm and the acetal carbon atom C-2 ($\delta_C$ = 102.2 ppm) were seen.

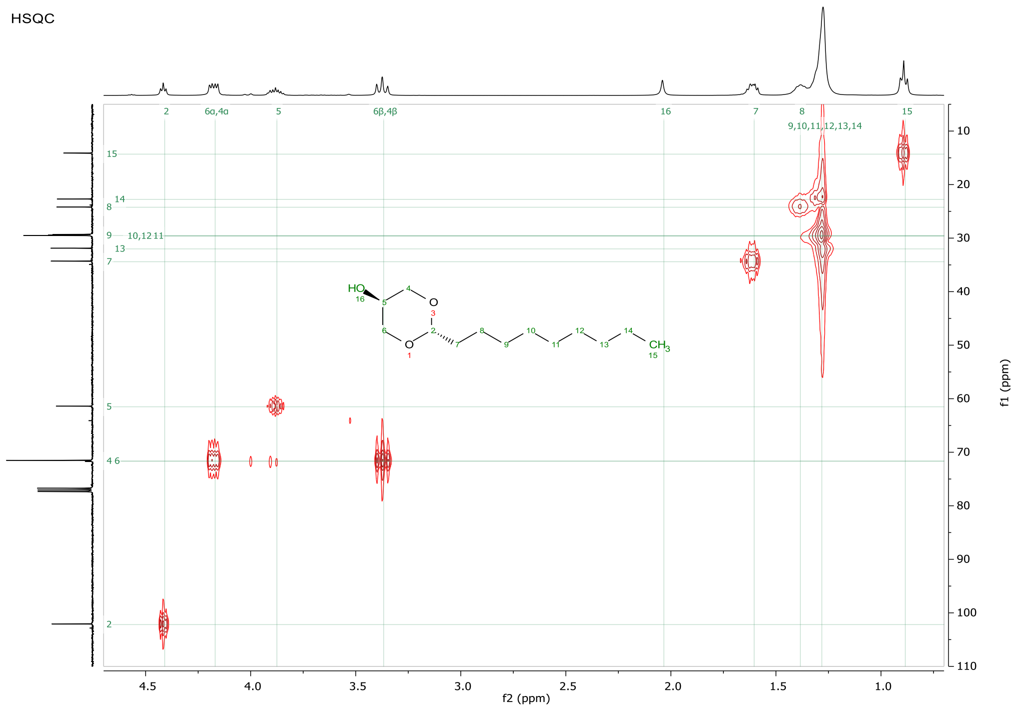


*Figure B. HSQC experiment on the solid separated from **18a** ($CDCl_3$, 25°C).*

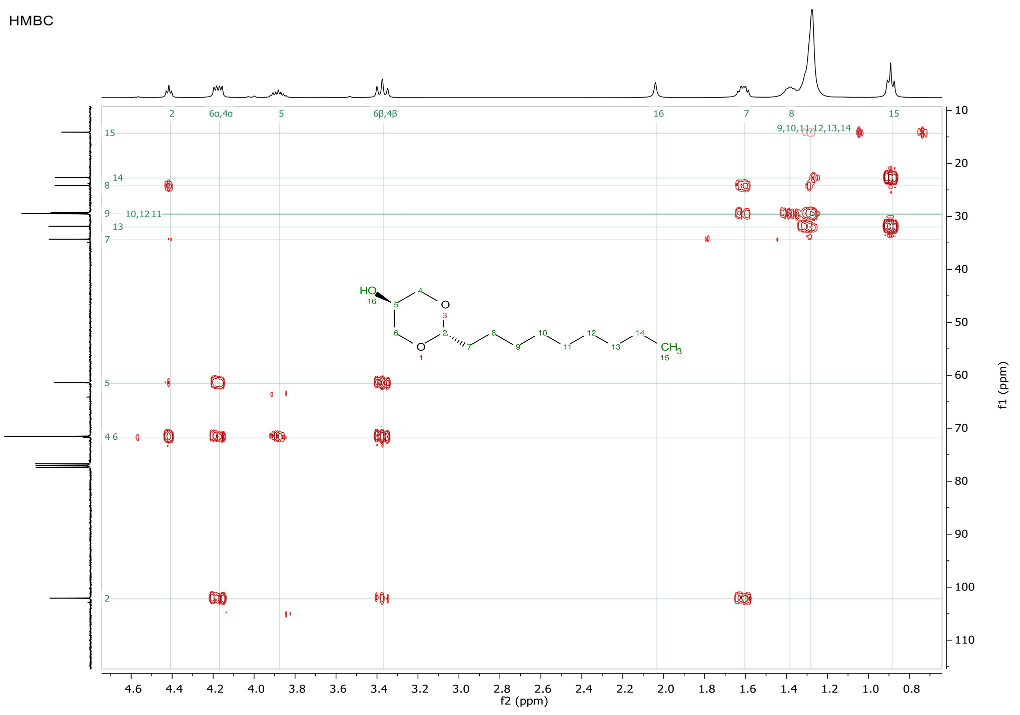

*Figure C. HMBC experiment on the solid separated from 18a (CDCl3, 25°C).*

The whole set of data above was consistent with the anticipated 1,3-dioxane structure **18a.6** (Figure D), where the occurrence of some proton and carbon nuclei as enantiotopic pairs ($H^{\alpha}$-4/$H^{\alpha}$-6, $H^{\beta}$-4/$H^{\beta}$-6, and C-4/C-6, respectively) explains the observed coupling and intensity patterns. By contrast, the alternative 1,3-dioxolane connectivity (**18a.5**) can be ruled out with confidence because matching with the recorded data would require the very unlikely, accidental isochronism within each of three pairs of not-equivalent nuclei (C-5/C-6 and, *e.g.*, $H^{\alpha}$-5/$H^{\alpha}$-6 and $H^{\beta}$-5/$H^{\beta}$-6).

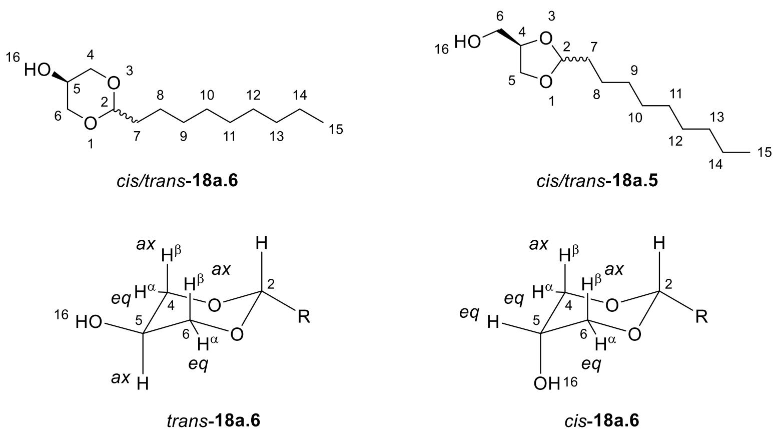


*Figure D.* Structures and numbering schemes of the 1,3-dioxane and 1,3-dioxolane isomers (**18a.6** and **18a.5**, respectively) of the reaction products between glycerol and decanal and most stable chair-like conformers of the *trans* and *cis* diastereomers of **18a.6**.

Having established the atom connectivity as **18a.6**, the relative configuration at its stereogenic units was examined next. A first clue towards this goal came from the analysis of the multiplet of H-5 at $\delta_H$ = 3.86 ppm, whose rather large coupling constants ($^{3}J_A$ = 10.2 Hz and $^{3}J_B$ = 5.0 Hz) identified *ax-ax* and *ax-eq* relationships between the proton under exam and those located at the vicinal methylene positions.

Following previous reports on closely related substances,[32, 34] when the most stable ψ-chair conformers are considered the likely candidate structure was *trans*-**18a.6** rather than *cis*-**18a.6** (Figure D). Additional proof of this conclusion was sought by nuclear Overhauser effect (nOe) measurements. The experiments, carried out by selective irradiation of the proton nuclei within the heterocycle core and the hydroxy group, revealed through-space $^{1}H$-$^{1}H$ dipolar interactions in accordance with the structure *trans*-**18a.6** and the proposed proton assignments (Figure E).

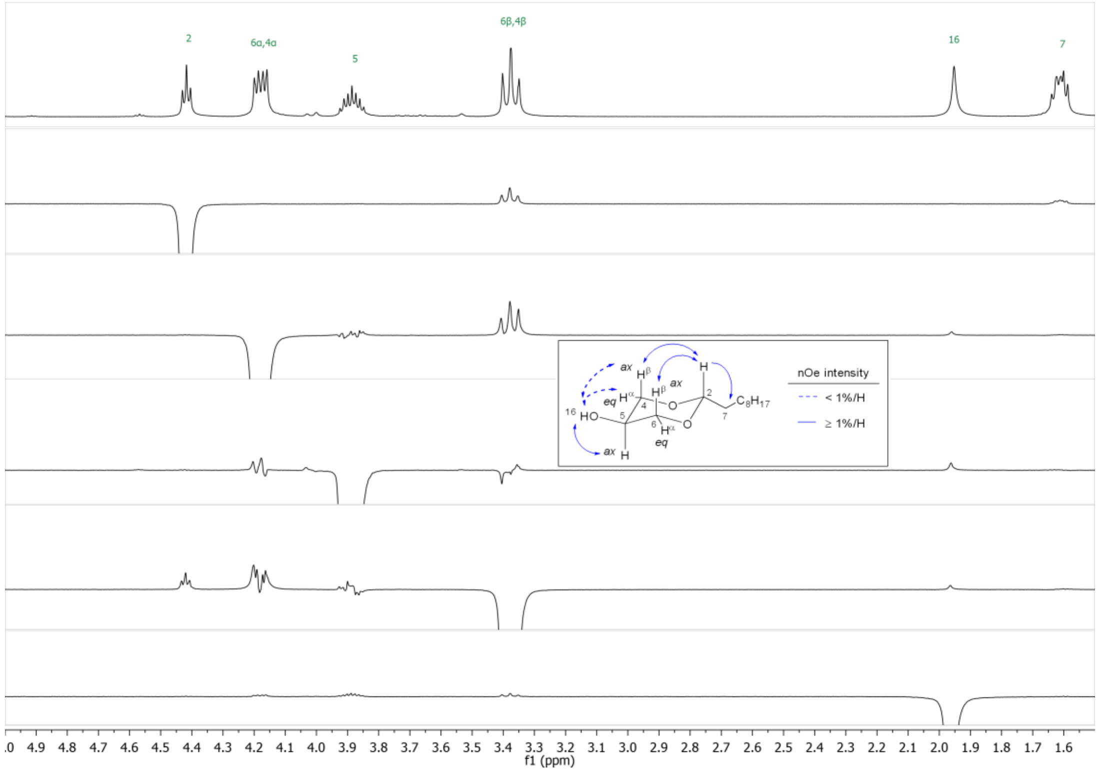

Figure E. nOe experiments by irradiation of selected resonances of the solid separated from **18a** ($CDCl_3$, 25°C).

Overall, the conclusions above mirror those of Tian et al. for a 5-hydroxy-1,3-dioxolane unit embed within iridoid glucoside dimers from *Dipsacus asper*.[36] Moreover, the good matching of the spectroscopic constants ($\delta$ and $J$) of relevant $^{1}$H and $^{13}$C nuclei with those reported in the literature for other long-chain *trans*-2-alkyl-5-hydroxy-1,3-dioxolanes,[33-34] lend support the somewhat more empirical assignments carried out in the previous studies.

It is worth of note that, upon standing overnight, further resonances began to appear in the spectra of the sample in $CDCl_3$. The chemical shift values of the new signals matched those observed with whole product mixture **18a** (Figure F). Based on the $^{13}$C NMR data reported for the analogous 2-pentadecyl derivatives,[33] such additional lines are tentatively assigned to the diastereomer (*cis*-**18a.6**) and ring isomers (*cis*- and *trans*-**18a.5**) of the initially separated solid component (Figure G).

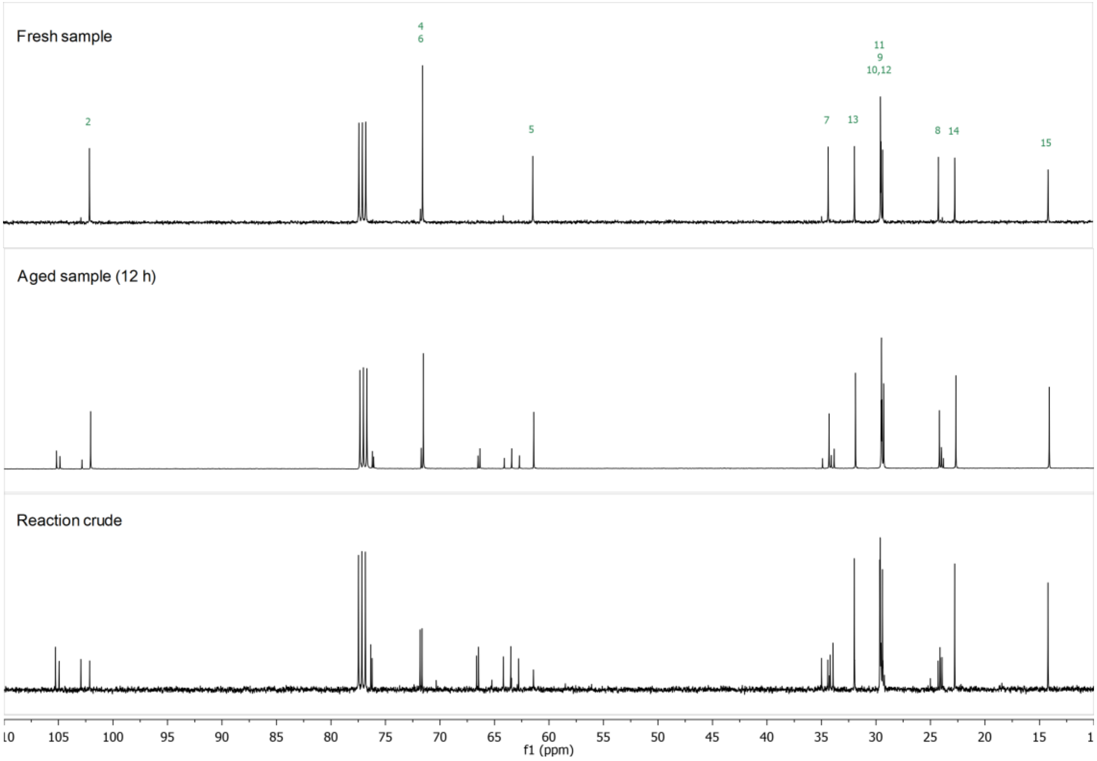


*Figure F. Comparison between the $^{13}$C NMR spectra ($CDCl_3$, 25°C) of a freshly prepared (top) and aged $CDCl_3$ solution (middle) of the solid separated from **18a** and a freshly prepared solution of the whole product mixture **18a** (bottom).*

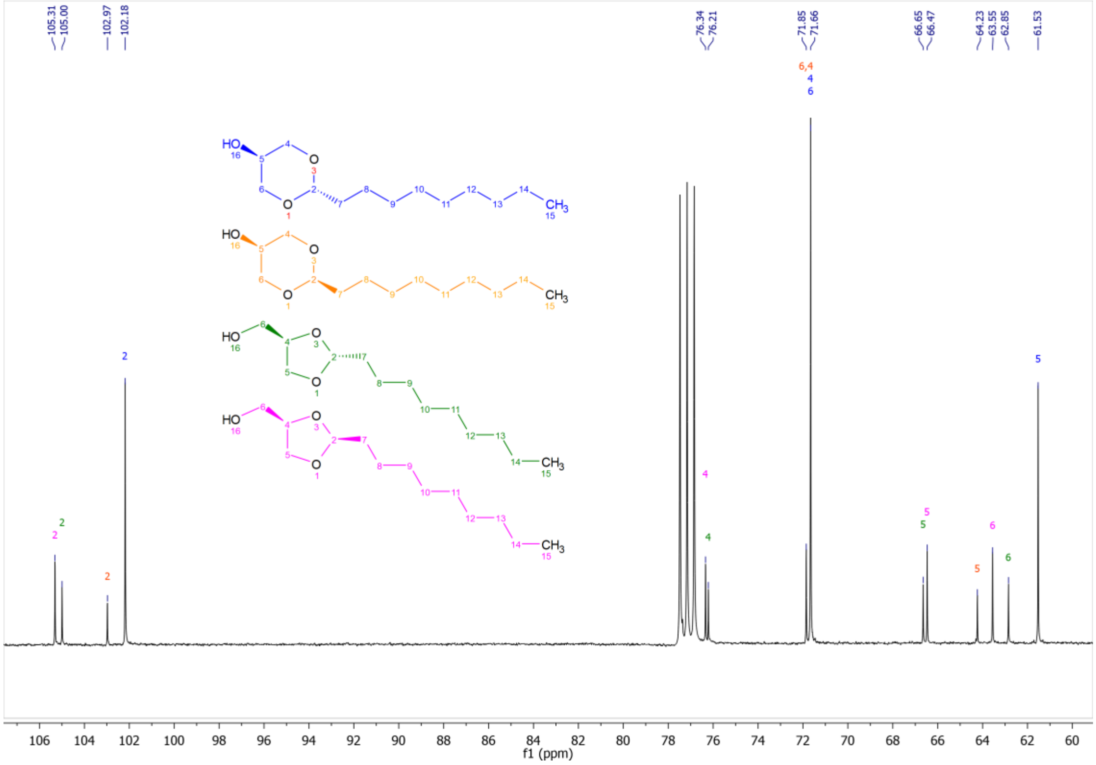


*Figure G. Resonances of oxygenated carbon atoms and proposed assignments in the $^{13}$C NMR spectrum of the aged sample of figure F ($CDCl_3$, 25°C).*

## Prediction of some physicochemical properties of the cyclic hydroxyacetals.

Table B summarizes the total number of carbon atoms ($n_c$) of the repellents examined in this study, together with their octanol-water partition coefficient (*logP*), polar surface area (*PSA*), and saturated vapour pressure (*logVP*), as predicted by the *ChemBrain IXL 5.9* database and computation software.

The program may be found at www.neouronix.ch. The atom-additive computation methods and parameters employed for predicting *logP* and *logVP* have been described by Naef and co-workers.[37-38]

Due to the lack of enough database entries, structurally related to ketone-derived acetals, the *logVP* estimates could be obtained only for aldehyde-derived products.

*Table B. Selected physicochemical descriptors of DEET (1), Icaridin (2), and the hydroxyacetal repellents examined in this study.*

| Code | Structure | $n_C$ [a] | *logP* [b] | *PSA* [c] | *logVP* [d] |
|---|---|---|---|---|---|
| **1** | 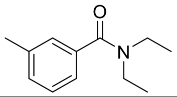 | 12 | 2.57 | 21 | -0.77 |
| **2** | 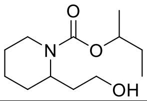 | 12 | 2.03 | 46 | -2.85 |
| **05b** | 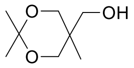 | 8 | 2.12 | 37 | - |
| **06b** | 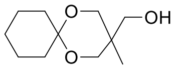 | 11 | 3.04 | 37 | - |
| **07b** | 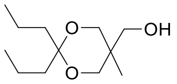 | 12 | 3.56 | 38 | - |
| **07c** | 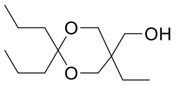 | 13 | 3.92 | 32 | - |
| **09a** | 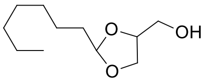 | 11 | 1.71 | 43 | -0.44 |
| | 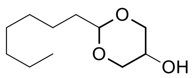 | | 2.19 | 43 | -0.06 |
| **09b** | 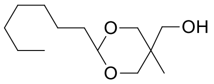 | 13 | 2.27 | 37 | -1.1 |
| **10a** | 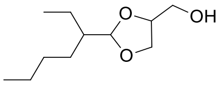 | 11 | 1.65 | 41 | -0.17 |
| | 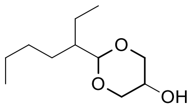 | | 1.65 | 41 | 0.21 |
| **11a** | 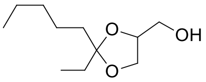 | 11 | 3.36 | 41 | - |
| **12a** | 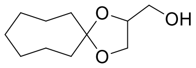 | 11 | 3.20 | 38 | - |

| | | | | | |
|---|---|---|---|---|---|
| **12b** | 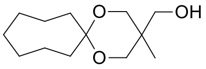 | 13 | 3.76 | 33 | - |
| **13a** | 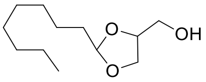 | 12 | 2.07 | 43 | -0.91 |
| | 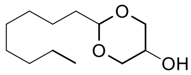 | | 2.07 | 42 | -0.53 |
| **14a** | 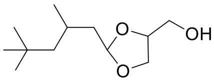 | 12 | 1.95 | 43 | -0.22 |
| | 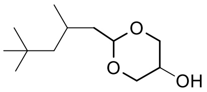 | | 1.95 | 43 | 0.16 |
| **15a** | 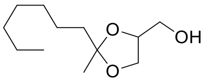 | 12 | 3.72 | 41 | - |
| **16b** | 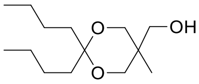 | 14 | 4.29 | 30 | - |
| **16c** | 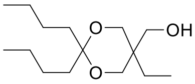 | 15 | 4.64 | 37 | - |
| **17a** | 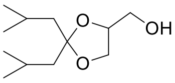 | 12 | 3.60 | 36 | - |
| | 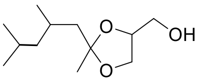 | | 3.60 | 38 | - |
| **17b** | 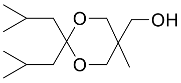 | 14 | 4.16 | 33 | - |
| | 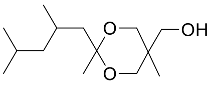 | | 4.16 | 36 | - |
| **17c** | 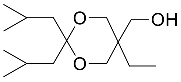 | 15 | 4.52 | 31 | - |
| | 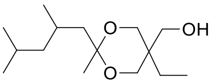 | | 4.52 | 37 | - |
| **18a** | 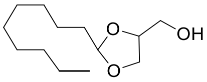 | 13 | 2.43 | 42 | -1.38 |
| | 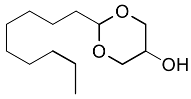 | | 2.43 | 42 | -1.00 |
| **19a** | 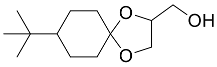 | 13 | 3.80 | 42 | - |
| **20a** | 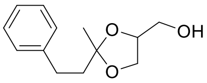 | 13 | 3.56 | 42 | - |
| **21a** | 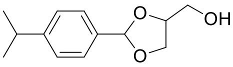 | 13 | 1.85 | 42 | -1.17 |
| | 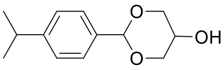 | | 1.85 | 41 | -0.79 |
| **22a** | 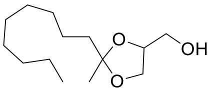 | 14 | 4.44 | 40 | - |
| **23a** | 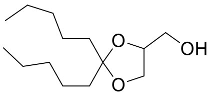 | 14 | 4.44 | 37 | - |

| | | | | | |
|---|---|---|---|---|---|
| **24a** | 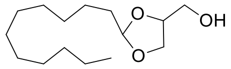 | 15 | 3.15 | 42 | -2.32 |
| | 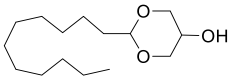 | | 3.15 | 42 | -1.94 |
| **25a** | 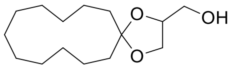 | 15 | 4.64 | 39 | - |

[a] Total number of carbon atoms in the substance.

[b] Logarithm to the basis 10 of the predicted octanol-water partition coefficient.

[c] Logarithm to the basis 10 of the predicted polar surface area in $A^2$.

[d] Logarithm to the basis 10 of the predicted saturated vapour pressure at 25°C, in Pascal.

## References


1. Matsukizono, H.; Matsumoto, K.; Endo, T., Multifunctional Cyclic Carbonates Comprising Hyperbranched Polyacetals: Synthesis and Applications to Polymer Electrolytes and Networked Polymer Materials. *J. Polym. Sci., Part A: Polym. Chem.* **2019,** *57* (23), 2295-2303.
2. Raskildina, G. Z.; Spirikhin, L. V.; Zlotskij, S. S.; Kuznetsov, V. V., Conformational Analysis of 5-Ethyl-5-hydroxymethyl-2,2-dimethyl-1,3-dioxane. *Russ. J. Org. Chem.* **2019,** *55* (4), 502-507.
3. Huang, J.-W.; Chen, C.-D.; Leung, M.-K., Magnesium bromide promoted Barbier-type intramolecular cyclization of halo-substituted acetals, ketals, and ortho esters. *Tetrahedron Lett.* **1999,** *40* (49), 8647-8650.
4. Kouklovsky, C., Product class 8: 1,3-dioxanes, 1,3-dioxepanes, and larger-ring O/O acetals. *Sci. Synth.* **2007,** *29*, 487-612.
5. Nurberdiev, R.; Yazkuliev, O.; Khekimov, Y. K.; Zlotskii, S. S.; Rakhmankulov, D. L., Synthesis of alkyl and formyl(hydroxymethyl)-substituted dioxospirans. *Izv. Akad. Nauk Turkm. SSR, Ser. Fiz.-Tekh., Khim. Geol. Nauk* **1990,** (6), 101.
6. Nurberdyev, R. U.; Khekimov, Y. K.; Yazkulyev, O.; Berdimyradov, N.; Akhmedova, S.; Khaidarov, R. M., Synthesis of (2,2-dialkyl-1,3-dioxacycloalkylmethyl) formates. *Izv. Akad. Nauk Turkm. SSR, Ser. Fiz.-Tekh., Khim. Geol. Nauk* **1990,** (5), 109.
7. Crotti, C.; Farnetti, E.; Guidolin, N., Alternative intermediates for glycerol valorization: iridium-catalyzed formation of acetals and ketals. *Green Chem.* **2010,** *12* (12), 2225-2231.
8. Piasecki, A., Acetals and ethers. XXI. Preparation of diastereomerically pure sodium salts of sulfated 2-n-alkyl-5-hydroxymethyl-5-methyl-1,3-dioxanes. *Synth. Commun.* **1992,** *22* (3), 445.
9. Sokolowski, A.; Piasecki, A.; Burczyk, B., Chemical structure and surface activity. XXV. Synthesis and surface properties of chemodegradable anionic surfactants: sodium salts of sulfated 2-n-alkyl-5-hydroxymethyl-5-methyl-1,3-dioxanes. *J. Am. Oil Chem. Soc.* **1992,** *69* (7), 633.
10. Boekelheide, V.; Liberman, L.; Figueras, J.; Krespan, C.; Pennington, F. C.; Tarbell, D. S., Drugs effecting muscular paralysis. Some substituted dioxolanes and related compounds. *J. Am. Chem. Soc.* **1949,** *71*, 3303.
11. Taillandier, G.; Domard, M.; Boucherle, A., Application of Verloop's parameters. Comparison with other steric parameters and selection problems. *Farmaco, Ed. Sci.* **1980,** *35* (2), 89-109.
12. Anteunis, M.; Borremans, F.; Gelan, J.; Heyndrickx, L.; Vandenbroucke, W., N.M.R. experiments on ketals. XI. Shifts of acyclic and cyclic ketals. *Bull. Soc. Chim. Belges* **1967,** *76* (9-10), 533.
13. Pawar, R. R.; Gosai, K. A.; Bhatt, A. S.; Kumaresan, S.; Lee, S. M.; Bajaj, H. C., Clay catalysed rapid valorization of glycerol towards cyclic acetals and ketals. *RSC Adv.* **2015,** *5* (102), 83985-83996.
14. Stefanovic, D.; Petrovic, D., Structure of glycerol acetals. *Tetrahedron Lett.* **1967,** (33), 3153.
15. Norinder, J.; Rodrigues, C.; Boerner, A., Tandem hydroformylation-acetalization with a ruthenium catalyst immobilized in ionic liquids. *J. Mol. Catal. A: Chem.* **2014,** *391*, 139-143.

16. Grayson, S. M.; Frechet, J. M. J., Synthesis and Surface Functionalization of Aliphatic Polyether Dendrons. *J. Am. Chem. Soc.* **2000,** *122* (42), 10335-10344.
17. Marine, J. E.; Liang, X.; Song, S.; Rudick, J. G., Azide-rich peptides via an on-resin diazo transfer reaction. *Biopolymers* **2015,** *104* (4), 419-426.
18. Gaziola, L.; Bornscheuer, U.; Schmid, R. D., A rapid and effective separation of enantiomers of glycerol derivatives by gas chromatography and their lipase-catalyzed biotransformation. *Enantiomer* **1996,** *1* (1), 49-54.
19. Ceita, L.; Gavina, P.; Lopez Lavernia, N.; Llopis, C.; Mestres, R.; Tortajada, A., Polymer-supported acetals as systems for protection and controlled delivery of volatile aldehydes. *React. Funct. Polym.* **1996,** *31* (3), 265-272.
20. Gorbunov, D. N.; Shchukina, T. V.; Kardasheva, Y. S.; Sinikova, N. A.; Maksimov, A. L.; Karakhanov, E. A., Cation-exchange resins in the hydroformylation-acetalization tandem reaction. *Pet. Chem.* **2016,** *56* (8), 711-716.
21. Woelfel, K.; Hartman, T. G., Mass spectrometry of the acetal derivatives of selected generally recognized as safe listed aldehydes with ethanol, 1,2-propylene glycol and glycerol. *ACS Symp. Ser.* **1998,** *705*, 193-210.
22. Gatzi, K.; Muller, P. Spirocyclic esters of sulfurous acid as pesticides. US2730529, 1956.
23. Kheifits, L. A.; Moldovanskaya, G. I.; Gol'dovskii, A. E.; Il'ina, G. P., Ethylene ketals of some alkylcyclohexanones. *Zh. Vses. Khim. O-va. im. D. I. Mendeleeva* **1964,** *9* (6), 706.
24. Vigh, G.; Estrada, I. R. T. Fluorescence labeling reagents and uses thereof. US20130224870, 2013.
25. Vigh, G.; Estrada, R. T. Fluorescence labeling reagents and uses thereof. WO2012027717, 2012.
26. Maryanoff, B. E.; Nortey, S. O.; Gardocki, J. F.; Shank, R. P.; Dodgson, S. P., Anticonvulsant O-alkyl sulfamates. 2,3:4,5-Bis-O-(1-methylethylidene)-β-D-fructopyranose sulfamate and related compounds. *J. Med. Chem.* **1987,** *30* (5), 880.
27. Broquet, C.; Auclair, E.; Blavet, N.; Touvay, C.; Braquet, P., Aminoacylates and aminocarbamates of 2-substituted 4-hydroxymethyl-1,3-dioxolanes as ammonium salts. A new series of PAF antagonists. *Eur. J. Med. Chem.* **1990,** *25* (3), 235.
28. McDougall, P. J. Lubricating composition containing 1,3-dioxolane-4-methanol compounds as antiwear additives. WO2012071154, 2012.
29. Walker, J.; Saveliev, S.; Zeng, Z.; Kargbo, R.; Wang, H.; Levin, S.; Ressler, V.; Zhang, J.; Shi, C.; Uyeda, H. T.; Osterman, J.; Zhou, M.; Rai, P.; Zhou, W. Acid-labile surfactants for protein sample preparation. WO2021026316, 2021.
30. De Wit, P. P.; Van der Steeg, M.; Van der Gen, A., Remarkable electrophilic properties of the pentaenol ether system of fecapentaene-12. *Tetrahedron Lett.* **1986,** *27* (51), 6263.
31. Piasecki, A.; Burczyk, B.; Sokolowski, A.; Kotlewska, U., Acetals and ethers. Part XXII. An efficient method for the preparation of pure long-chain cis- and trans-2-n-alkyl-5-hydroxy-1,2-dioxanes. *Synth. Commun.* **1996,** *26* (22), 4145-4151.
32. Baumann, W. J., Configuration and conformation of the long-chain cyclic acetals of glycerol. *J. Org. Chem.* **1971,** *36* (19), 2743.
33. Wedmid, Y.; Evans, C. A.; Baumann, W. J., Synthesis of cyclic glycerol acetal phosphates: proton and carbon-13 NMR characteristics of isomeric 1,3-dioxolane and 1,3-dioxane phosphate structures. *J. Org. Chem.* **1980,** *45* (9), 1582.
34. Piasecki, A.; Soko-lowski, A.; Burczyk, B.; Gancarz, R.; Kotlewska, U., Synthesis, Surface Properties, and Hydrolysis of Chemodegradable Anionic Surfactants: Diastereomerically Pure Sodium cis- and trans-2-n-Alkyl-1,3-dioxan-5-yl Sulfates. *Langmuir* **1997,** *13* (6), 1434-1439.
35. Kautz, J.; Feltrin, W.; Sales, E. S.; Eifler-Lima, V. L.; Merlo, A. A., Condensation reaction of glycerol and carbonyl compounds: synthesis, characterization, and derivatization for liquid crystals. *Quim. Nova* **2015,** *38* (8), 1053-1062.
36. Tian, X.-Y.; Wang, Y.-H.; Liu, H.-Y.; Yu, S.-S.; Fang, W.-S., On the chemical constituents of Dipsacus asper. *Chem. Pharm. Bull.* **2007,** *55* (12), 1677-1681.
37. Naef, R. A Generally Applicable Computer Algorithm Based on the Group Additivity Method for the Calculation of Seven Molecular Descriptors: Heat of Combustion, LogPO/W, LogS, Refractivity,

Polarizability, Toxicity and LogBB of Organic Compounds; Scope and Limits of Applicability *Molecules (Basel, Switzerland)* [Online], 2015, p. 18279-18351.

38. Naef, R.; Acree, W. E., Jr., Calculation of the Vapour Pressure of Organic Molecules by Means of a Group-Additivity Method and Their Resultant Gibbs Free Energy and Entropy of Vaporization at 298.15 K. *Molecules (Basel, Switzerland)* **2021,** *26* (4).

## Table S1. Structure and repellent properties of all synthesized hydroxyacetals against *A. albopictus*.

| ***Compound*** | ***Code*** | ***No. C*** | ***100% PE (µg/cm²)*** | ***Average PT (min) >95% (% coefficient of variation)**** | ***Estimated 100% PT (min, mean±SE)**** |
|---|---|---|---|---|---|
| DEET | 01 | | 8.3 | 120 (1.4) | 120±26 |
| Icaridin | 02 | | 8.3 | 480 (2.2) | 370±52 |
| 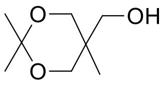 | 05b | c08 | | 0 | |
| 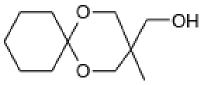 | 06b | c11 | 0.83 | 480 (3.3) | 375±89 |
| 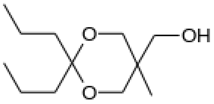 | 07b | c12 | | 300 (1.4) | 220±87 |
| 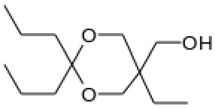 | 07c | c13 | | 360 (3.3) | 60±34 |
| 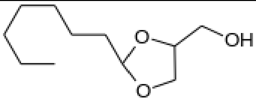 | 09a | c11 | 8.3 | 420 (4.5) | 307±53 |
| 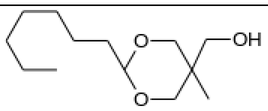 | 09b | c13 | | 420 (5.6) | 120±105 |
| 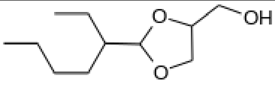 | 10a | c11 | | 360 (56.8) | |
| 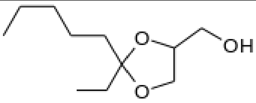 | 11a | c11 | | 240 (3.8) | 345±66 |
| 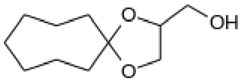 | 12a | c11 | 1.7 | 480 (0) | 380±20** |
| 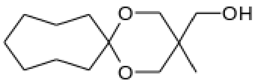 | 12b | c13 | | 420 (3.1) | 240±60 |
| 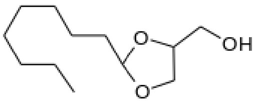 | 13a | c12 | | 420 (4.7) | 270±77 |
| 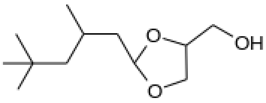 | 14a | c12 | | 360 (4.5) | |

| | | | | | |
|---|---|---|---|---|---|
| 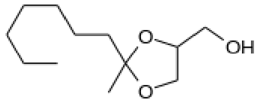 | 15a | c12 | 16.7 | 480 (6.0) | 380±61 |
| 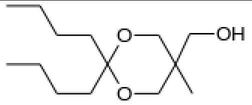 | 16b | c14 | 1.7 | 420 (8.0) | 180±64 |
| 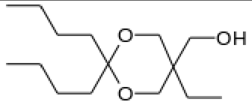 | 16c | c15 | 1.7 | 480 (1.4) | |
| 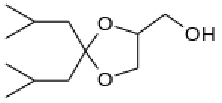 | 17a | c12 | | 300 (2.3) | |
| 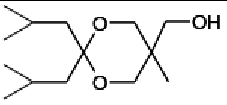 | 17b | c14 | 8.3 | 480 (5.0) | 300±94** |
| 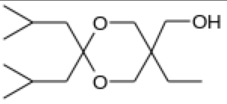 | 17c | c15 | 8.3 | 480 (0) | |
| 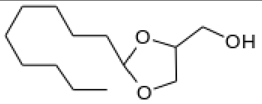 | 18a | c13 | 8.3 | 420 (2.1) | 190±50 |
| 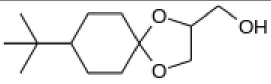 | 19a | c13 | | 0 | |
| 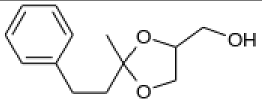 | 20a | c13 | | 0 | |
| 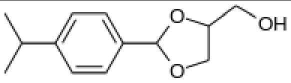 | 21a | c13 | | 0 | |
| 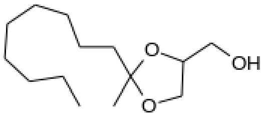 | 22a | c14 | 8.3 | 420 (3.5) | 380±105 |
| 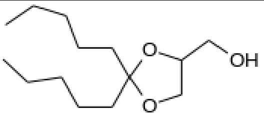 | 23a | c14 | 83.3 | 360 (4.8) | 240±65 |
| 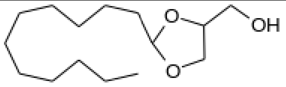 | 24a | c15 | | 0 | |
| 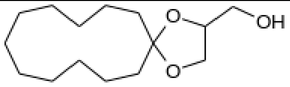 | 25a | c15 | | 0 | |

*at the dose of 0.17mg/$cm^2$

**For 12a and 17b, compounds best performing in terms of repellency and toxicity, the estimated Complete protection time was compared with DEET; Log Rank Mantel, *p<0.05 (by considering Bonferroni correction).

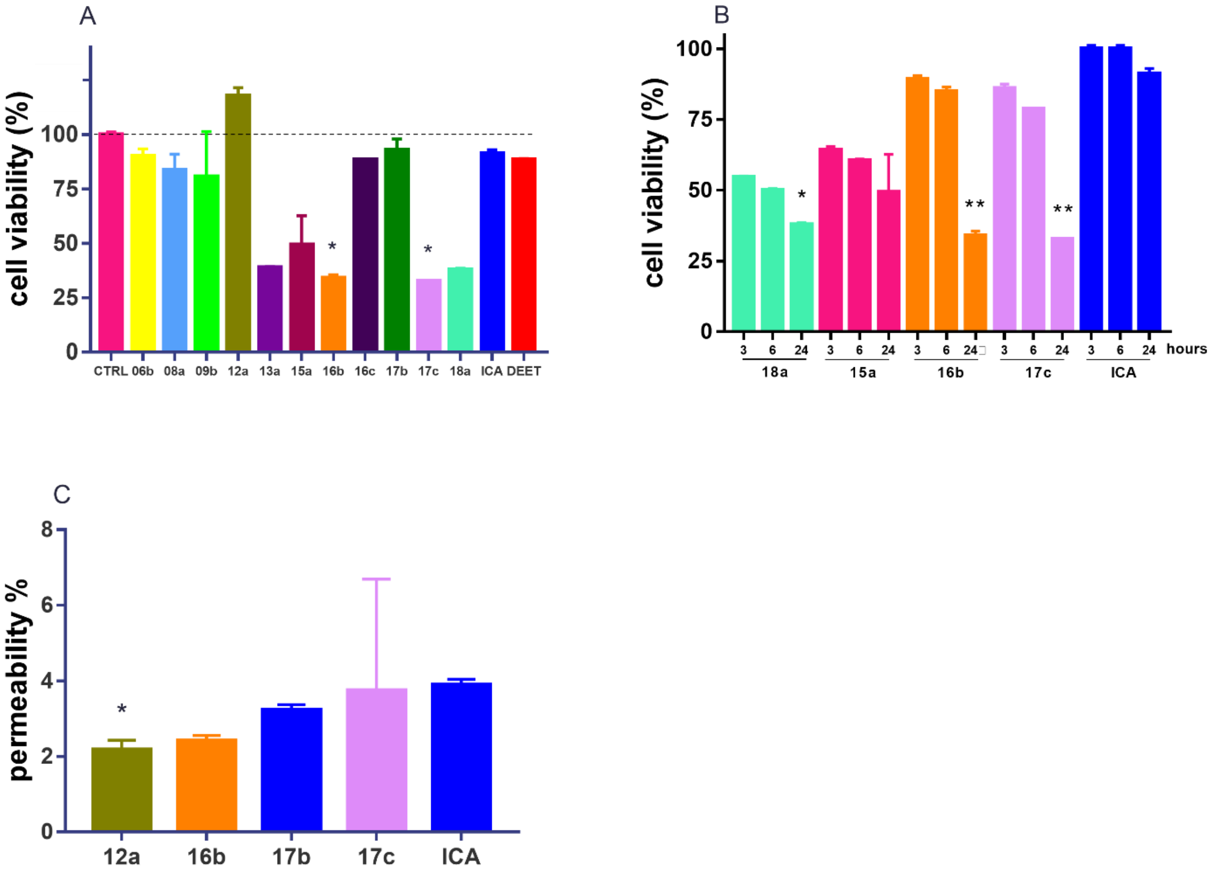


**Fig. S1**. (A) Cytotoxicity on normal human keratinocytes (HaCaT) exposed to eleven of the synthesized compounds, Icaridin (ICA) or DEET tested at 82 µg/ml for 24 h; (B) Cytotoxicity on HaCaT cells exposed to four of the synthesized compounds and ICA after 3, 6 and 24 h; (C) percentage of the compounds passed through a Caco2 cell monolayer in a transwell permeation test. *p <0.05 and **p<0.01 vs ICA by Kruskal-Wallis test and Dunnett's Multiple Comparisons test; data are expressed as mean ± SE of three independent experiments.
